# Preparation and Properties of Asymmetric Polyvinyl Pyrroli-Done/Polycaprolactone Composite Nanofiber Loaded with Tea Tree Extract

**DOI:** 10.3390/polym14183714

**Published:** 2022-09-06

**Authors:** Yu Xu, Lei Xie, Tingting Hou, Di Wang, Tong Zhang, Chengpeng Li

**Affiliations:** 1 School of Chemistry and Environmental Science, Guangdong Ocean University, Zhanjiang 524088, China; 2 Zhanjiang Key Laboratory of Comprehensive Utilization of Chemical Research for Marine Resources, Zhanjiang 524088, China; 3 Zhanjiang Key Laboratory of Marine Bio-Materials Research, Zhanjiang 524088, China

**Keywords:** nanofiber, polyvinyl pyrrolidone, antibacterial activity, antioxidant activity

## Abstract

To develop a novel asymmetric nanofiber membrane with antioxidant and antibacterial activities, biocompatible polyvinyl pyrrolidone (PVP) and polycaprolactone (PCL) were used as carriers to load water-soluble tea tree extract (TTE) and hydrophobic tea tree oil (TTO) via electrospinning and electrostatic spraying, respectively, which was named as TTE-PVP-3/TTO-PCL. The results show that uniform TTE-PVP nanofibers with an average diameter of 95 ± 27 nm could be obtained when the mass ratio of TTE to PVP was set as 1:3. Homogeneous TTO/PCL microspheres with an average size of 4.38 ± 0.79 µm could be obtained when the propulsion speed was 0.08 mm/min and the voltage was 10 KV. The activity study showed that TTE could only improve the antioxidant activity of TTE-PVP-3/TTO-PCL, while TTO could improve the antibacterial activity effectively. Under experimental conditions, the inhibition zones of TTE-PVP-3/TTO-PCL against *Staphylococcus aureus* and *Escherichia coli* were 7.50 ± 0.48 mm and 9.55 ± 0.45 mm, respectively, and its scavenging rates for 2,2-diphenylpicrylhydrazyl (DHPP) and hydroxyl radical were 59.79 ± 4.10% and 61.02 ± 4.95%, respectively. In conclusions, TTE-PVP-3/TTO PCL can be potentially used as a new kind of anti-oxidative and antibacterial wound dressings.

## 1. Introduction

Electrospinning by means of high-voltage electrostatic action provides a strategy to produce nanofibers from a variety of polymer materials [1]. Under the effects of surface tension, polymer solutions or melts in a spinneret inclines to reduce their exposed surface and become a sphere [2]. When the electrostatic repulsion of the charged polymer liquid is higher than the surface tension, a conical shape known as Taylor’s cone is formed and the jet initiation starts from the cone tip [3]. Due to the strong cohesive force in the polymer solutions or melts, a stable jet is ejected and nanofibers are produced under the stretching [4]. Nanofibers, ranging from 5 to 500 nm, possess high specific surface area and high porosity, and their topology closely mimics that of the native extracellular matrix [5]. Thus, nanofibers are widely applied as wound dressings [3,6]. On the other hand, infection caused by pathogenic microorganisms severely delay the healing of wounds [7,8]. During the period of the inflammatory stage, great levels of free radicals were produced by neutrophils, leucocytes, and monocytes, resulting in the disorder of cellular mechanism and the further delaying of wound-healing [9,10]. Therefore, a couple of functional nanofiber dressings with antioxidant and antibacterial activities have been developed recently [11,12,13].

Due to good biocompatibility, polyvinyl pyrrolidone (PVP) was initially used as a plasma substitute during World War II [14]. In the past twenty years, PVP has been widely used in the development of nanofiber wound dressings, scaffolds, hydrogels, and tissue engineering as well [15,16]. However, deficiency in antioxidant and antibacterial activities has greatly limited PVP applications in wound-healing. A previous study showed that natural green tea extract loaded in PVP nanofiber resulted in greatly improved antioxidant activities [13] and antibacterial activities [12]. Thus, the exploration of new active natural products is of great importance. The original distribution area of the Australian tea tree, also named *Melaleuca alternifolia*, is in the subtropical costal region of New South Wales in Australia [17]. In the last twenty years, the Australian tea tree has been successfully cultured in other similar climatic zones, including China. Tea tree oil (TTO) is mainly the liposoluble extracts from the leaves and branches of the Australian tea tree, which contains approximately 100 compounds, mainly monoterpenes, sesquiterpenes, and their alcohol derivatives [18]. According to reports, its major components include terpinen-4-ol, γ-terpinene, α-terpinene, α-terpineol, α-terpinolene, 1,8-cineole, etc. [19], which showed good activity against bacteria and fungi [20,21,22]. For instance, Koseki and co-author found that TTO can effectively inhibit *C. albicans* and that certain concentrations of TTO can remove its biofilm attached to resin using lower levels of abrasion [22]. Meanwhile, research found that three terpenic compounds α-terpinene, α-terpinolene, and γ-terpinene in TTO endowed it with strong antioxidant performance [21]. Therefore, TTO has been introduced in various polymer films or nanofibers such as chitosan [23], polylactic acid [24], poly(ethylene oxide) [25], and polyurethane [20] for functionalization. Recently, Hu and co-authors fabricated several asymmetric chitosan films with a hydrophilic chitosan/silk fibroin repair layer and hydrophobic composite layers using the solution casting method [23]. The hydrophobic composite layers contained a bacteriostatic TTO layer and antioxidant sodium ascorbate-entrapped poly (lactic-co-glycolic acid) microspheres, which can relieve infection and induce antioxidant response for skin repair effectively [23]. However, no asymmetric nanofibers or films of PVP with antioxidant and antibacterial activities have been reported before.

Herein, novel asymmetric composite nanofibers containing a continuous PVP layer and a discontinuous polycaprolactone (PCL) layer were fabricated by electrospinning and electrostatic spraying, respectively, wherein the hydrophilic PVP nanofibers were the carrier materials for the water-soluble extracts of the Australian tea tree and hydrophobic polycaprolactone (PCL) microspheres were the carriers for TTO. The fabrication parameters, microscopic morphology, antioxidant activities, and antibacterial performance of the asymmetric composite nanofibers were evaluated systematically. To our best knowledge, it was the first report on asymmetric composite nanofibers containing both the water-soluble extracts and liposoluble extracts of the Australian tea tree. We expected that the asymmetric composite nanofibers may supply guidance for the development of the new generation of wound dressings with antioxidant and antibacterial activities.

## 2. Materials and Methods

### 2.1. Materials

Australian tea tree leaves were supplied by Dashan Seedling Farm (Zhangpu County, Fujian, China). Tea tree oil was obtained from Jiangxi Kangshengtang Pharmaceutical Industry Co., Ltd. (Ji’an, China). Polyvinylpyrrolidone (PVP, M_W_ = 1,300,000), polycaprolactone (PCL, M_W_ = 14,000), 1,1-diphenyl-2-picryl-hydrazyl (DHPP), and ferrous sulfate heptahydrate (AR) were all supplied by Shanghai Macklin Biochemical Co., Ltd. (Shanghai, China). Hydrogen peroxide solution (30 wt%) was purchased from Xilong Chemical Co., Ltd. (Shantou, China). Anhydrous ethanol (AR) was supplied by Sinopharm Chemical Reagent Co., Ltd., (Shanghai, China). Potassium ferricyanide (AR), ferric chloride hexahydrate (AR), and trichloroacetic acid (AR) were provided by Guangdong Guanghua Technology Co., Ltd., (Guangzhou, China). *Escherichia coli* (*E. coli*, CMCC(B) 44103) and *Staphylococcus aureus* (*S. aureus*, ATCC 25923) were all obtained from China Center of Industrial Culture Collection (Beijing, China). Tryptic soy agar (TSA) and tryptone soya broth (TSB) were obtained from Beijing Land Bridge Technology Co., Ltd., (Beijing, China).

### 2.2. Water Extraction

A total of 8.5 g of Australian tea tree leaves (ATTL) were sealed in a cellulose thimble, which was then placed in a Soxhlet apparatus. ATTL was then refluxed using deionized water (180 mL) as the solvent for two hours. After filtration, the as-obtained tea tree extracts (TTE) solution was concentrated with a final solid content of 3.21 ± 0.15 wt% and stored for later use. The element composition of the solid extracts was tested using Elementar Vario (EL cube, Heraeus, Germany) using Dumas’s combustion method. Carbon and nitrogen elements in samples were firstly converted into CO_2_ and N_2_, respectively, which were then separated and detected using a thermos-conductive detector. Three parallel tests were repeated, and an average was used as the final results. Based on the results, the detailed compositions for the solid extracts were carbon (40.9%), oxygen (39.9%), nitrogen (0.65%), and sulfur (0.17%).

### 2.3. Electrospinning and Electro-Spraying

PVP (20 g) was firstly dissolved in deionized water (80 g) in a 60 °C water bath to obtain a 20 wt% solution. The TTEA solution obtained in 2.2 was then mixed with PVP solution (20 wt%) under magnetic stirring at weight ratios of 1:5, 1:4, and 1:3, which were named electrospinning solution 1, 2, and 3. The electrospinning solutions were then transferred in a 2.5 mL syringe. Based on the preliminary optimization, electrospinning was carried out under 16 kV voltage, a 14 cm tip-to-collector distance, 0.09 mm min^−1^ flow rate, 60 ± 5% relative humidity, and 25 ± 1 °C. The nanofibers obtained from electrospinning solutions 1, 2, and 3 were named TTE-PVP-1, TTE-PVP-2, and TTE-PVP-3, respectively. For comparison, neat PVP nanofiber was also fabricated and named PVP-0.

For electro-spraying, a PCL solution (25 wt%) was firstly prepared using TTO as the solvent. The PCL solution was then transferred in a 5.0 mL syringe and electro-sprayed under 10 KV voltage, a 10 cm tip-to-collector distance, 0.06~0.09 mm min^−1^ flow rate, 60 ± 5% relative humidity, and 25 ± 1 °C using TTE-PVP-1, TTE-PVP-2, or TTE-PVP-3 as collector. After electrospraying for twenty minutes, samples obtained using TTE-PVP-1, TTE-PVP-2, and TTE-PVP-3 as collector were named TTE-PVP-1/TTO-PCL, TTE-PVP-2/TTO-PCL, and TTE-PVP-3/TTO-PCL, respectively. For comparison, the sample electrosprayed for forty minutes with TTE-PVP-3 as a collector was named TTE-PVP-3/TTO-PCL-2.

### 2.4. Characterizations

Nanofiber morphology was investigated using a scanning electron microscope (SEM, Tesscan Mira Lms, Brno, Czech Republic) after gold coating. The average nanofiber diameters were calculated by measuring 100 nanofibers randomly selected from each sample and analyzing them via Nano Measurer 1.2 software (Fudan University, Shanghai, China). The Fourier transform infrared spectrum (FTIR) was recorded using a spectrometer (TENSOR 27, Bruker, Karlsruhe, Germany) under attenuated total reflection mode. The scanning wavenumber was in the range of 4000 and 600 cm^−1^, with a resolution of 4 cm^−1^.

### 2.5. Antioxidative Capacity Evaluation

DPPH free radical scavenging assay

The free radical scavenging activities of samples were measured in terms of hydrogen donating or radical scavenging ability using the DPPH method with slight modification [26]. Briefly, 0.2 mmol L^−1^ DPPH solution was prepared using anhydrous ethanol (Shanghai Macklin Biochemical Co., Ltd., Shanghai, China) as solvent. The TTEA solution (2 mL) was then mixed with the DPPH solution (2 mL), which was then vortexed and incubated for 0.5 h at room temperature. The absorbance of the mixture at 518 nm was spectrophotometrically tested after incubation. The decreased absorbance indicated an increase of the DPPH radical scavenging activity. For nanofiber evaluation, a 0.06240 g fiber sample (around 20 mm × 20 mm) was dispersed in 2 mL PBS solution (pH = 6.6, GIBCO BRL, Grand Island, NY, USA) under vortexing, which was then mixed with a DPPH solution (2 mL) following the same procedure. For comparison, a control solution using a PBS solution (2 mL) to replace the sample solution and a blank solution using the mixture of a PBS solution (2 mL) and an ethanol solution (2 mL) were also prepared and spectrophotometrically measured. The scavenging activity of the DPPH free radical was calculated using the following formula:

DPPH scavenging effect (%) = (A_1_ + A_3_ − A_2_)/A_1_ × 100

where A_2_, A_3_, and A_1_ were the absorbance of the solution with the treated sample, the solution with the blank sample, and the solution without samples.

Hydroxyl radical scavenging activity assay

Hydroxyl radicals were generated by a Fenton reaction, and the scavenging activity was measured according to previous report with minor changes [26,27]. The reaction mixture consisted of a TTE solution (2 mL), 6 mmol L^−1^ FeSO_4_ solution (2 mL), and 6 wt% H_2_O_2_ solution (2 mL). After vortexing for ten minutes. a 6 mmol L^−1^ salicylic acid solution (2 mL) was then added under vortexing. The total mixture (8.0 mL) was incubated at 35 °C for 30 min, and the absorption value recorded as A_4_. Then the deionized water was used to replace the developer H_2_O_2_ solution, and the corresponding absorption value was defined as A_5_. The deionized water was also substituted for the sample solution, and its absorbance was recorded as A_6_. For nanofiber samples, a 0.06240 g fiber sample (around 20 mm × 20 mm) was dispersed in a 2 mL PBS solution to obtain a nanofiber solution, which was then treated following the same procedure. The absorbance at 514 nm was recorded, and the scavenging activity was calculated as follows:

Hydroxyl radical scavenging rate (%) = 100 × (A_6_ + A_5_ − A_4_)/A_6_

The experimental data were statistically analyzed and expressed as a mean ± standard deviation (SD). Statistical differences were determined using one-way ANOVA with IBM SPSS Statistics 22, and the statistical significance was set at *p* < 0.05.

### 2.6. Antibacterial Assay

Two pathogenic bacteria, *S. aureus* and *E. coli*, were selected to evaluate the potential applications in wound infection. Prior to inhibition zone tests, an aliquot containing 100 µL of a 107 CFU mL^−^^1^ bacterial suspension was spread on the surface of nutrient agar plates. Nanofiber membranes with a diameter of 6 mm were then placed on the agar surface. After incubation for 24 h at 37 °C, the growth inhibition zones were measured using a vernier caliper. The final results were averaged based on three parallel experiments.

## 3. Results and Discussion

### 3.1. Characterization

#### 3.1.1. Morphology

**Figure 1:**
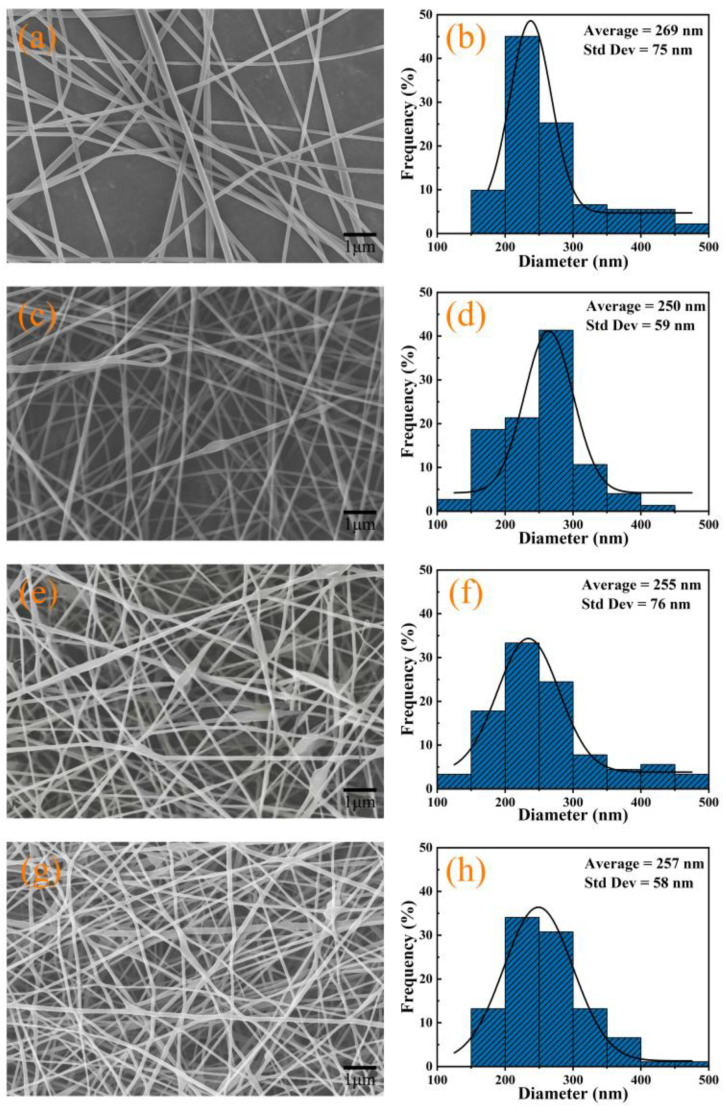
SEM images and fiber diameter distributions for nanofibers: (**a**,**b**) PVP-0; (**c**,**d**) TTE-PVP-1; (**e**,**f**) TTE-PVP-2; (**g**,**h**) TTE-PVP-3.

Figure 1 shows that uniform, random distributed nanofibers could be obtained under the optimized electrospinning conditions. Based on the statistic results, the average diameters for PVP-0, TTE-PVP-1, TTE-PVP-2, and TTE-PVP-3 were 285 ± 77 nm, 117 ± 27 nm, 104 ± 25 nm, and 95 ± 27 nm, respectively. It is clear that the average fiber diameter in Figure 1 decreases with the increased loading of TTE, which coincides well with the PVP composite nanofibers containing green tea extract [13]. With the increased content of TTE, the weight fraction of PVP in the electrospinning solution decreases, and the corresponding viscosity diminishes as well. Thus, the degree of PVP chain entanglement will decrease, leading to the decreased diameter [28].

**Figure 2:**
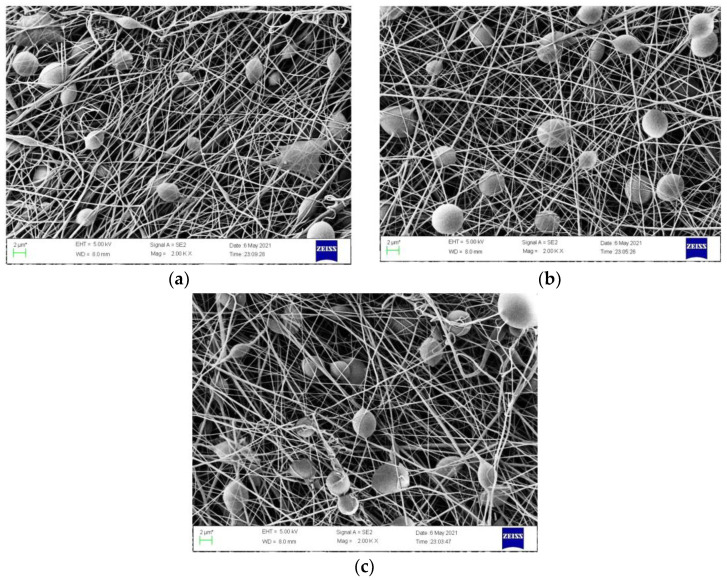
SEM photos show the effects of the flow rate on the morphology of the TTO/PCL microspheres in TTE-PVP-3/TTO-PCL after electrospinning for two minutes: (**a**) 0.07 mm min^−1^, (**b**) 0.08 mm min^−1^, (**c**) 0.09 mm min^−1^.

Figure 2 shows the TTO/PCL microspheres produced under the different flowing rates. It shows that unstable jet-flow was produced at a low flow rate. When the flow rate was 0.07 mm min^−1^, deformed microspheres were produced. When the flow rate was increased to 0.08 mm min^−1^, homogenous microspheres with an average diameter of 4.38 ± 0.79 μm (measured by Nano Measure1) were obtained. When the flow rate reached 0.09 mm/min, the microspheres became deformed or slightly adhesive, which may be due to the delayed evaporation [29]. Therefore, the flow rate was set to 0.08 mm min^−1^.

#### 3.1.2. Chemical Structure

**Figure 3:**
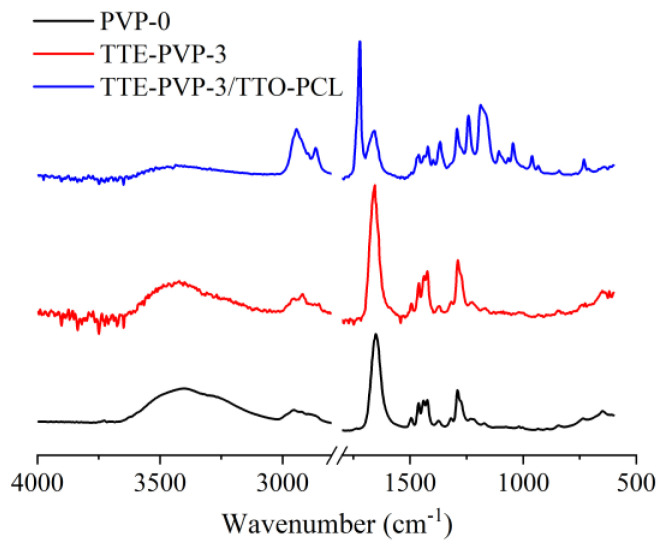
FTIR results for pure PVP-0, TTE-PVP-3, and TTE-PVP-3/TTO-PCL.

As can be seen from Figure 3, the absorption peaks at 2956 cm^−1^, 1676 cm^−1^, and 1232 cm^−1^ corresponded to the characteristic absorption bands of the C-H bond, C=O bond, and C-N bond in PVP membranes, respectively [30]. No significant changes were observed in the absorption of TTE-PVP-3 compared to the pure PVP-0. After the loading of TTO-PCL microspheres, a strong peak located at 1722 cm^−1^ was emerged in the TTE-PVP-3/TTO-PCL, which was assigned to the C=O stretching vibration of the ester bond in PCL [31]. Furthermore, the peaks appeared at 1107 cm^−1^, 1045 cm^−1^, and 732 cm^−1^ were C-O stretching, C-O-C symmetric stretching, and the rocking vibration of (CH_2_)_n_ (n ≥ 4), which attributed to the characteristic signals of TTO. Thus, it can be demonstrated that the asymmetric polyvinyl pyrrolidone/polycaprolactone composite nanofibers have been successfully fabricated.

### 3.2. Antioxidant Properties

**Figure 4:**
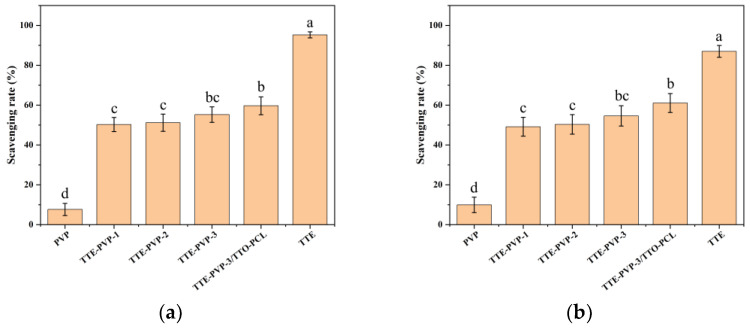
Scavenging capacities for (**a**) DPPH and (**b**) hydroxyl free radicals. Values are given as mean ± SD from three independent replications; different lowercase letters on the bars show significant difference at *p* < 0.05.

Free radical scavenging capacity is the primary indicator for the antioxidant activity. As shown in Figure 4a, the DPPH free radical scavenging rate for the pure PVP-0 was only 7.83 ± 3.00%, which was significantly lower than all other groups (*p* < 0.01). With the enhanced loading of TTE, the DPPH free radical scavenging rate showed an increased inclination, but there were no significant differences among TTE-PVP-1, TTE-PVP-2, and TTE-PVP-3. According to reference, terpenic compounds such as α-terpinene, α-terpinolene, and γ-terpinene in TTO possess good antioxidant activity [21]. Our findings showed that the DPPH free radical scavenging rate for TTE-PVP-3/TTO-PCL was significantly higher than for TTE-PVP-1 and TTE-PVP-2, but there were no significant differences between TTE-PVP-3 and TTE-PVP-3/TTO-PCL. It is worth mentioning that pure TTE possessed the strongest DPPH free radical scavenging rate in spite of the loading of TTE and TTO/PCL microspheres (*p* < 0.01) On the other hand, the scavenging rate of the hydroxyl free radical showed the same inclination as that of DPPH free radical. Thus, it can be concluded that TTE loading is very useful for the improvement of antioxidant activity. Based on the previous report, the non-volatile compositions from the Australia tea tree were mainly 3,3′-O-dimethylellagic acid and pentacyclic triterpenes [32], but no references about the TTE have been reported. Based on our element analysis, the oxygen content is nearly equal to that of carbon. Thus, it can be deduced that TTE may contain water-soluble compounds with carboxyl groups or hydroxyl groups, which possess good antioxidant activity.

### 3.3. Antibacterial Properties

**Figure 5:**
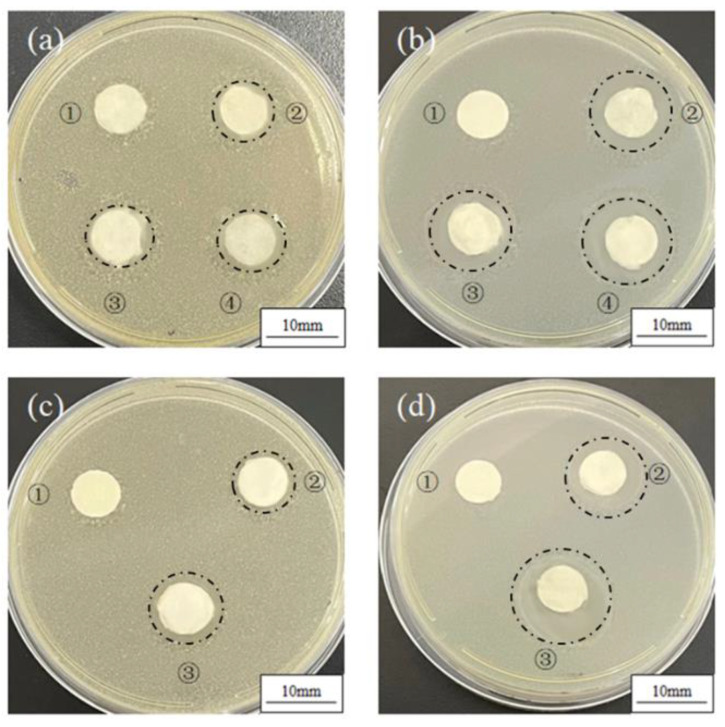
Inhibition zone of *S. aureus* (**a**) and *E. coli* (**b**)*:* ①, ②, ③, and ④ refers to TTE-PVP-3, TTE-PVP-3/TTO-PCL, TTE-PVP-2/TTO-PCL, and TTE-PVP-1/TTO-PCL; effects of electrostatic spraying time on the inhibition zone against *S. aureus* (**c**) and *E. coli* (**d**)*:* ①, ②, and ③ refers to TTE-PVP-3, TTE-PVP-3/TTO-PCL, and TTE-PVP-3/TTO-PCL-2, respectively.

As can be seen from Figure 5a,b, TTE-PVP-3 showed no clear antibacterial activity for both *S. aureus* (a) and *E. coli*. On the other hand, TTE-PVP-3, TTE-PVP-2, and TTE-PVP-1 loaded with the same dosage of TTO-PCL microspheres possessed the same antibacterial activity for both *S. aureus* (7.51 ± 0.50 mm) and *E. coli* (11.08 ± 0.65 mm). Thus, it can be concluded that the mass content of TTE and PVP did not affect the bacterial inhibition performance of the nanofiber membranes. Figure 5c,d showed that the effect of TTO-PCL microsphere dosage on the antibacterial activity of TTAE-PVP-3 with electrospraying times of 0 min, 20 min, and 40 min were 0 mm, 7.50 ± 0.48 mm, and 9.55 ± 0.45 mm for *S. aureus*, respectively, while those for *E. coli* were 0 mm, 11.16 ± 0.50 mm, and 14.52 ± 0.30 mm, respectively. To sum up, antibacterial activity is determined by the TTO loading.

## 4. Conclusions

The TTE-PVP-3/TTO-PCL composite nanofiber films were prepared by electrostatic spinning and electrostatic spraying techniques using hydrophilic TTE and hydrophobic TTO as model active substances, and PVP and PCL as carriers. The average diameter of TTE-PVP-3 nanofibers was about 95 ±28 nm, and the average diameter of TTO-PCL micrometer spheres was 4.38 ± 0.79 µm. The activity study showed that TTE could effectively scavenge DPPH and hydroxyl radicals, but the antibacterial activity of TTE was not significant. The inhibition circles of TTE-PVP-3/TTO-PCL were 9.55 ± 0.45 mm and 14.52 ± 0.30 mm for *Staphylococcus aureus* and *Escherichia coli*, respectively, under experimental conditions. The above findings suggest that TTE-PVP-3/TTO-PCL can be potentially applied as a new type of wound dressing. Further research will explore the detailed composition of the TTE, as well its antioxidant mechanism.

## Data Availability

The data presented in this study are contained within the article.
